# The efficacy of recombinant tissue plasminogen activator in severe post-operative fibrinous reaction after uneventful phacoemulsification - a case series and review

**DOI:** 10.22336/rjo.2022.7

**Published:** 2022

**Authors:** Ainul Basirah Ibramsah, Aliff Irwan Cheong, Wan Norliza Wan Muda, Mohtar Ibrahim

**Affiliations:** * Department of Ophthalmology, Hospital Tengku Ampuan Afzan, Pahang, Malaysia; ** Department of Ophthalmology and Visual Sciences, School of Medical Sciences, Universiti Sains Malaysia, Kelantan, Malaysia

**Keywords:** fibrin, fibrinolysis, phacoemulsification

## Abstract

**Objective:** To report the effectiveness of recombinant human tissue plasminogen activator (rtPA) in severe post-operative fibrinous reaction.

**Method:** The presentation of a case series.

**Result:** Four patients developed severe post-operative inflammation about six days after an otherwise uneventful cataract surgery. Three women, with an average age of 63.25 years old, were included. At the same time, three patients had underlying comorbidities such as diabetes mellitus and hypertension. The average duration of the operation was 31.25 minutes. All operations were performed by different surgeons. All underwent uneventful cataract surgery. They presented with a dense fibrin in anterior chamber within a week of post-operative review. All patients received 25 micrograms in 0.1 mL of intracameral rtPA injection. Assessment included anterior chamber fibrin reaction before and after injection by slit lamp biomicroscopy two hours, 24 hours and one week after rtPA application. Serial visual acuity and intraocular pressure (IOP) were taken pre and one-week post rtPA application. Injection of rtPA effectively caused fibrinolysis in all the cases presented.

**Conclusion:** Fibrinolysis after cataract surgery with conventional topical medications can be time consuming and less efficient. Intracameral application of 25 μg rtPA is an efficient management of fibrin reaction in cataract surgery.

**Abbreviations:** rtPA = recombinant tissue plasminogen activator, IOP = intraocular pressure, BCVA = best corrected visual acuity, PCIOL = posterior chamber intraocular lens

## Introduction

Fibrinous reaction is a serious undesirable consequence of post cataract surgery that may lead to visual loss. The standard anti-inflammatory agents used to treat post-operative inflammation are usually inadequate to cause fibrinolysis. The aim of this study was to evaluate the efficacy of recombinant human tissue plasminogen activator (rtPA) in fibrinolysis, after cataract surgery. 

Fibrin is a tough protein substance that is arranged in long fibrous chains; it is formed from fibrinogen, a soluble protein that is produced by the liver and found in blood plasma. Inflammatory response associated with cataract surgery is due to disruption of the blood-aqueous barrier and by the release of inflammatory mediators. Prostaglandins are released from the iris and ciliary body during cataract surgery, especially with manipulation of the iris. Change in blood aqueous barrier causes an increase in vascular permeability, stimulating platelet activation, platelet-derived growth factor secretion and increased levels of fibrinogen. Fibrinogen is not visible with slit lamp examination, but will appear in the anterior chamber when converted to fibrin [**1**-**3**].

Fibrin formation may lead to multiple potential complications that include pain, photophobia, posterior synechiae, pseudophakic cellular precipitates, uveitis, elevated intraocular pressure and glaucoma, if left untreated [**4**]. 

## Methods

In this case series, four eyes of four patients who developed fibrin reaction after uneventful phacoemulsification surgery received 25 micrograms (mcg) in 0.1 milliliters (mL) of intracameral rtPA injection. Patients were evaluated for anterior chamber fibrin reaction before and after injection by slit lamp biomicroscopy two hours, 24 hours and one week after rtPA application. Intraocular pressure (IOP) was taken by Goldmann applanation tonometry. Best corrected visual acuity (BCVA) was tested using a standard Snellen chart before and one week after rtPA application.

## Results

Patient A was a 70-year-old female, with underlying diabetes mellitus and hypertension. She had no ocular comorbidity and underwent uneventful right eye phacoemulsification and posterior chamber intraocular lens (PCIOL) implant. She developed severe inflammation with fibrinous reaction on day six of post-operation and vision dropped from 6/ 12 to 6/ 60. She received intracameral rtPA injection on day 12 post-operative and her BCVA improved to 6/ 9, two days later.

Patient B was a 69-year-old female, with no known medical illness or ocular comorbidity. She underwent uneventful left eye phacoemulsification and PCIOL implant. Minimal Descemet tear was observed at the main wound and paracentesis, otherwise the procedure was uneventful. The duration of the operation was 30 minutes. She developed fibrinous anterior chamber reaction on day five post-operation and received intracameral rtPA injection three days later. Her pre-operative vision was counting fingers, 6/ 18 during the inflammation, and improved to 6/ 9 after receiving intracameral rtPA injection.

**Fig. 1:**
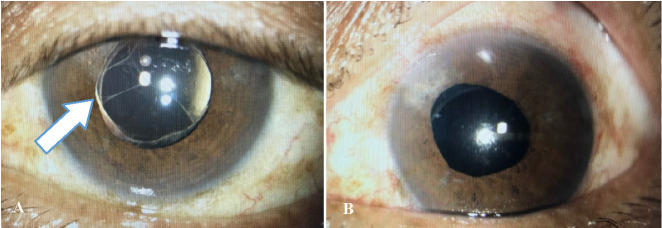
**A.** Arrow showing fibrin formation on day 10 post-operation in Patient C. **B.** Resolved fibrin post intracameral rtPA injection

Patient C was a 64-year-old man, with underlying diabetes mellitus, hypertension, and bilateral proliferative diabetic retinopathy. He underwent uneventful left phacoemulsification and PCIOL implantation but the duration of operation was slightly longer - 40 minutes due to poor pupil dilatation. He developed severe inflammation with fibrinous reaction on day 10 post-operation (**Fig. 1 A**) and received intracameral rtPA injection two days later (**B**). His BCVA improved from 6/ 60 to 6/ 9.

**Fig. 2:**
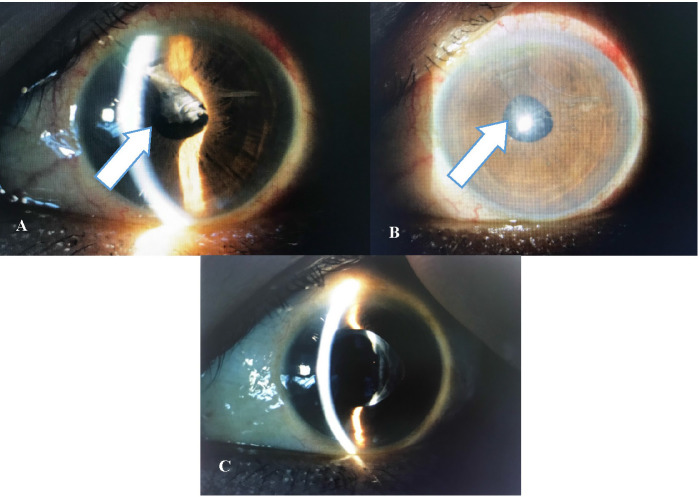
**A, B.** Arrow showing fibrin formation three days post operation in Patient D, **C.** Resolved fibrin post intracameral rtPA injection

Patient D was a 50-year-old female with multiple comorbidities such as diabetes mellitus, hypertension, and stage five chronic kidney disease. She had mild non-proliferative diabetic retinopathy in both eyes. Despite the uneventful left eye phacoemulsification with PCIOL implant, her vision did not improve. She developed a severe fibrinous reaction three days after surgery (**Fig. 2 A, B**). Intracameral rtPA administered the day after (**Fig. 2 C**) and BCVA improved from 6/ 60 to 6/ 9.

Surgeries were performed between July and August 2019, using standard procedure and equipment. The average duration of operation was 31.25 minutes. All the surgeries were performed by different surgeons. They were all implanted with acrylic one-piece PCIOL from various manufacturers.

All patients developed severe post-operative inflammation within one week after the surgery. Injection of rtPA effectively caused fibrinolysis in all the cases presented. No adverse events or ocular side effects such as hyphema, cornea toxicity or disturbance in IOP, were reported. The visual acuity improved in all patients with BCVA, being at least 6/ 9.

Results are summarized in **Table 1**.

**Table 1:** Summary of events and results for each patient

	Patient A	Patient B	Patient C	Patient D
Age	70	69	64	50
Comorbidity	Diabetes Mellitus, Hypertension	Nil	Diabetes Mellitus, Hypertension	Diabetes Mellitus, Hypertension, Chronic Kidney Disease stage 5
Ocular comorbidity	Nil	Nil	Bilateral Proliferative Diabetic Retinopathy	Mild Non- Proliferative Diabetic Retinopathy
Date of operation	22/ 7/ 19	23/ 7/ 19	25/ 7/ 19	5/ 8/ 19
Days of developing severe reaction post operation	6 days	5 days	10 days	3 days
Date of rTPA injection	3/ 8/ 19	3/ 8/ 19	3/ 8/ 19	17/ 8/ 19
Place of operation- Operation Theatre (OT)	OT A	OT A	OT B	OT B
Duration of operation	25 min	30 min	40 min	30 min
Type of procedure	Right phacoemulsification/ PCIOL	Left phacoemulsification/ PCIOL	Left phacoemulsification/ PCIOL	Left phacoemulsification/ PCIOL
Phacoemulsification machine	Brand A	Brand B	Brand C	Brand C
Viscoelastic	Brand D	Brand E	Brand F	Brand F
Intraocular lens	Brand G	Brand H	Brand G	Brand G
BCVA pre-operation	6/ 15	CF	6/ 60	6/ 60
BCVA pre-RTPA injection	6/ 60	6/ 18	6/ 6	6/ 60
BCVA final follow up	6/ 9	6/ 9	6/ 9	6/ 9
*Abbreviations: OT = operation theatre, PCIOL = posterior chamber intraocular lens, CF = counting fingers, BCVA = best corrected visual acuity*				

## Discussion

RtPA is a serine protease produced by recombinant technology. It is an enzyme that cleaves protein peptide bonds by catalyzing the conversion of plasminogen to plasmin [**5**]. It is widely used in thrombolysis of ischemic stroke, myocardial infarction, and pulmonary embolism [**5**]. In ophthalmology, rtPA is used as treatment of intraocular inflammation with fibrin formation and for failed blebs after glaucoma filtering surgery [**6**].

One of the identified risk factors for patients to develop post-operative inflammation, is diabetes. Studies show that diabetic patients have higher levels of flare in the aqueous fluid correlating with inflammatory markers, as compared to non-diabetic patients [**7**]. This may be associated with persistent hyperglycemia and a chronic systemic pro-inflammatory state. In our cases, three out of four patients were diabetic, hence explaining the hypothesis.

Internal and external audit were done with regards to the operation theatre, instruments used and other materials involved in the operation, however, no identifiable cause of severe inflammation was detected among these patients.

Extra caution needed to be taken in cases with neovascularization, iris surgical maneuvers, cataract extraction combined with trabeculectomy or vitrectomy as it could cause severe bleeding and hyphema, especially if rtPA was given immediately after surgery [**8**-**10**]. So far, no adverse events or ocular side effects were reported in our patients, until six months after intracameral rtPA injection.

## Conclusion

Severe immediate post uneventful cataract surgery fibrinous inflammation is a rare event. Early recognition of the condition is important to prevent complications. Treatment modality is by administration of rtPA. After cataract surgery, fibrinolysis with conventional topical medications can be time consuming and less efficient. Intracameral application of 25 μg rtPA is an efficient management of fibrin reaction in cataract surgery.

**Conflicts of Interest statement**

Authors have no conflict of interest.

**Informed Consent and Human and Animal Rights statement**

Informed consent has been obtained from all individuals included in this study.

**Authorization for the use of human subjects**

Ethical approval: The research related to human use complies with all the relevant national regulations, institutional policies, is in accordance with the tenets of the Helsinki Declaration, and has been approved by the review board of the School of Medical Sciences, Health Campus, Universiti Sains Malaysia.

**Acknowledgements**

Ms. Noor Osman for proofreading and editing the article.

**Sources of funding/ Financial support and sponsorship**

None.

**Disclosures**

None.

## References

[1] Heiligenhaus A, Steinmetz B, Lapuente R, Krallmann P, Althaus C, Steinkamp WK (1998). Recombinant tissue plasminogen activator in cases with fibrin formation after cataract surgery: a prospective randomised multicentre study. Br J Ophthalmol.

[2] Little HL (1981). Alterations in blood elements in the pathogenesis of diabetic retinopathy. Ophthalmology.

[3] Sebestyen JG (1982). Fibrinoid syndrome: a severe complication of vitrectomy surgery in diabetics. Ann Ophthalmol.

[4] Mohammadpour M, Jafarinasab MR, Javadi MA (2007). Outcomes of acute postoperative inflammation after cataract surgery. Eur J Ophthalmol.

[5] Jilani TN, Siddiqui AH (2021). Tissue Plasminogen Activator.

[6] Kristin N, McAlpine A, Owens T B, Trivedi R H, Perry LJP (2019). Evaluation of the etiology of persistent iritis after cataract surgery. J Ophthalmic Inflamm Infect.

[7] Tripathi RC, Tripathi BJ (2005). Tissue plasminogen activator therapy for the eye. Br J Ophthalmol.

[8] Dabbs CK, Aaberg TM, Aguilar HE, Sternberg P Jr., Meredith TA, Ward AR (1990). Complications of tissue plasminogen activator therapy after vitrectomy for diabetes. Am J Ophthalmol.

[9] Jaffe GJ, Abrams GW, Williams GA, Han DP (1990). Tissue plasminogen activator for postvitrectomy fibrin formation. Ophthalmology.

[10] Williams DF, Bennett SR, Abrams GW, Han DP, Mieler WF, Jaffe GJ, Williams GA (1990). Low-dose intraocular tissue plasminogen activator for treatment of postvitrectomy fibrin formation. Am J Ophthalmol.

